# Effect of High Myopia on Delayed Absorption of Subretinal Fluid after Scleral Buckling Surgery

**DOI:** 10.3390/jcm11133906

**Published:** 2022-07-05

**Authors:** Yongan Meng, Kejun Long, Jing Chen, Jing Luo

**Affiliations:** 1 Department of Ophthalmology, The Second Xiangya Hospital of Central South University, Changsha 410011, China; mengyongan@csu.edu.cn; 2 Hunan Clinical Research Center of Ophthalmic Disease, Changsha 410000, China; 3 Zhongshan Ophthalmic Center, Sun Yat-sen University, Guangzhou 510060, China; longkj@mail2.sysu.edu.cn (K.L.); chenj656@mail2.sysu.edu.cn (J.C.)

**Keywords:** scleral buckling, subretinal fluid, high myopia, optical coherence tomography

## Abstract

This study compared the absorption of subretinal fluid (SRF) in patients with rhegmatogenous retinal detachment (RRD) with and without high myopia after scleral buckling (SB) and investigated the effect of high myopia on SRF absorption. This retrospective study included patients with primary macula-off RRD grouped according to myopia and age. The optical coherence tomography (OCT) and OCT angiography indicators included subretinal fluid height (SRFH), subfoveal choroidal thickness (SFCT), and choroidal capillary blood flow density (CCFD) measured regularly. The presence of SRF 3 months after surgery was defined as delayed absorption. Overall, 90 eyes of 89 patients were enrolled, and 46 eyes (51.11%) had high myopia. In 43 eyes (47.78%), SRF absorption was delayed. There was no significant difference in SRF absorption after SB between the high and non-high myopia groups; younger patients (<35 years) had a higher probability of delayed absorption (*p* < 0.05). The SFCT in high myopia was significantly thinner than that in the non-high myopia group (*p* < 0.05); SFCT and SRFH were positively correlated (r_s_ = 0.275, *p* = 0.002), and there was a significant difference between the average CCFD with and without SRF (*p* < 0.05). High myopia had no significant effect on SRF absorption after SB.

## 1. Introduction

Rhegmatogenous retinal detachment (RRD) is an eye disease that causes blindness. Persistent subretinal fluid (SRF) refers to the presence of a small amount of subretinal fluid in the macular area detected in optical coherence tomography (OCT) after successful retinal reattachment in RRD patients. This condition is most common after scleral buckling (SB), and the reason for its occurrence and development is not clear [1]. Studies have shown that more than 50% of patients have persistent SRF after SB, compared to only 0–15% after pars plana vitrectomy (PPV) [2,3]. The mechanism of persistent SRF remains unclear: retinal pigment epithelium (RPE) pump dysfunction, decrease in choroidal blood flow, choice of surgical methods, and other factors may lead to persistent SRF after SB surgery. SRF in the macular area is the main factor leading to poor postoperative vision recovery. Therefore, studying its influencing factors can help guide the choice of surgical methods as well as predict and prevent SRF.

High myopia is a well-known risk factor for retinal breaks and RRD; the higher the degree of myopia, the higher the risk [4]. In myopia, a larger eyeball volume leads to RRD through earlier posterior vitreous detachment, common lattice degeneration, and a thinner retina than in emmetropia [5]. Michael et al. [1] explored the factors of persistent SRF after PPV and found that high myopia was a significant factor in the occurrence of SRF after RRD surgery. Therefore, the purpose of this study was to investigate whether patients with high myopia who underwent SB surgery were more likely to develop persistent SRF and the possible influencing mechanism of high myopia on delayed SRF absorption.

## 2. Materials and Methods

The subjects of this retrospective study were patients with primary macula-off RRD who had successfully undergone SB surgery in the Department of Ophthalmology, Second Xiangya Hospital, Central South University, from June 2018 to December 2020.

### 2.1. Data Collection

The inclusion criteria were: primary rhegmatogenous retinal detachment with an OCT examination showing retinal detachment involving the fovea; no other intraocular treatment during the follow-up period; and planned SB surgery. The exclusion criteria were: macular diseases; axial length (AL) greater than 30 mm or with posterior scleral staphyloma (in OCT/OCTA images of these eyes, the choroidal scleral interface was irregular, and the choroid was extremely thin, which made it impossible to accurately measure the relevant indicators, resulting in large errors in data analysis); retinal detachment due to ocular trauma or other eye diseases; retinal vascular diseases; history or presence of any other eye diseases that cause vision loss, such as glaucoma, strabismus, amblyopia, and optic nerve diseases; high-quality OCT images were not obtained during follow-up due to refractive medium turbidity; history of serious systemic diseases such as myocardial infarction, cerebral infarction, and liver and kidney dysfunction. According to the best scientific practice, we enrolled 90 eyes from 89 patients.

### 2.2. OCT&OCTA Assessment

All patients were examined for the best-corrected visual acuity with an ophthalmoscope, spectral-domain OCT (RTVue XR Avanti; Optovue, Inc., Fremont, CA, USA), Spectralis OCT (Heidelberg Engineering Company, Heidelberg, Germany), ocular B ultrasound, and ocular A ultrasound. All patients underwent standard SB surgery, including cerclage and padding. A postoperative indirect ophthalmoscopic examination showed reattachment of the retina.

The patients were followed up postoperatively for 7 days, 1 month, 3 months, 6 months, and 12 months. OCT and OCT angiography (OCTA) were performed during the follow-up, the disappearance of SRF was the endpoint of follow-up, and the presence of SRF 3 months after surgery was judged as delayed absorption. The primary outcome measure was the disappearance of subretinal fluid at 3 months postoperatively. The presence of SRF referred to the visible SRF during each follow-up, and non-SRF referred to the absence of SRF during each follow-up.

### 2.3. Primary Outcome Measures

**Figure 1:**
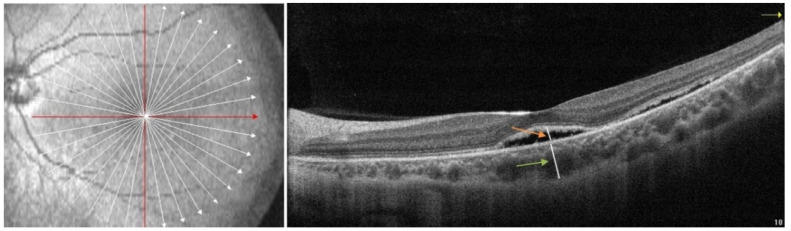
Measurements of the subfoveal choroidal thickness (SFCT) and subretinal fluid height (SRFH). The SFCT is the perpendicular distance from the outer boundary of the retinal pigment epithelium (RPE) layer to the inner border of the sclera (green arrow). The SRFH is the perpendicular distance from the outer boundary of the neuroepithelial layer to the inner border of the RPE layer (orange arrow).

**Figure 2:**
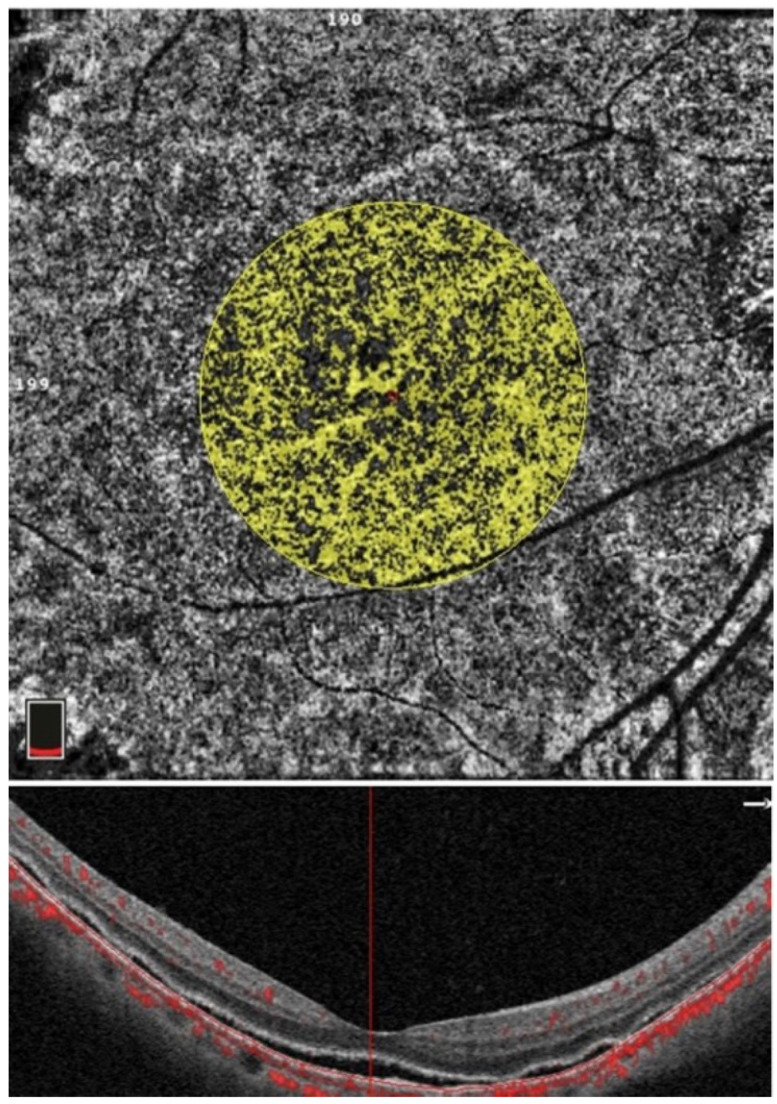
Measurement of the choroidal capillary blood flow density (CCFD). The flow area in the circle of 1.50 mm radius of choriocapillaris is measured, and the CCFD is obtained by the ratio of flow area to the selected area.

OCT and OCTA parameters were measured independently by two experienced doctors, and the measurement results were taken as the mean values. The measurements were repeated in the same way during follow-up. In the Optovue OCT, the macular area of the retina was scanned in radiation mode, and the subfoveal choroidal thickness (SFCT) and subretinal fluid height (SRFH) in the central fovea were manually measured using self-contained ruler software. The SFCT was the perpendicular distance from the outer boundary of the RPE layer to the inner border of the sclera (Figure 1, green arrow). The SRFH was the perpendicular distance from the outer boundary of the neuroepithelial layer to the inner border of the RPE layer (Figure 1, orange arrow). The choroidal macular region was scanned with Optovue HD Angio Retina mode, and the choriocapillaris flow density (CCFD) was calculated as the ratio of the flow area to the selected area of the choriocapillaris in the 3 × 3 mm range, centered on the macular fovea (Figure 2).

All patients were divided into the high myopia group (axial length ≥ 26.5 mm) or non-high myopia group (axial length < 26.5 mm), as well as the younger group (<35 years old) or the older group (≥35 years old). The delayed absorption of SRF was compared between the two groups after surgery.

### 2.4. Statistical Analysis

Statistical analyses were performed using IBM SPSS 26 software (IBM Corp., Armonk, NY, USA). The measurement data are indicated as mean ± SD; the absorption of SRF in the different groups was analyzed by Fisher’s exact test and binary logistic regression analysis; Spearman’s correlation was used to analyze the correlation between the SRFH and SFCT, and a two independent sample t-test was applied to compare the difference between CCFD with and without SRF.

## 3. Results

Overall, 90 eyes of 89 patients were included in this study: 42 eyes (46.67%) from males and 48 eyes (53.33%) from females; 37 eyes (41.11%) were younger than 35 years of age (mean ± SD: 22.70 ± 5.12 years), and 53 eyes (58.89%) were older than or equal to 35 years of age (mean ± SD: 53.32 ± 9.87 years). There was a total of 46 eyes (51.11%) with high myopia, the average AL was 26.95 ± 0.86 mm, and for 44 eyes (48.89%) without high myopia, the average AL was 23.75 ± 0.50 mm. SRF absorption was delayed in 43 eyes (47.78%), while it was completely absorbed in 47 eyes (52.22%) within 3 postoperative months (Table 1). 

There was no significant difference in SRF absorption between the high and non-high myopia groups after SB surgery (*p* = 0.408). The SRF absorption in the different age groups was statistically different; the probability of delayed SRF absorption in younger patients was higher than that in older patients (Table 2). Binary logistic regression analysis also showed that younger age was the sole independent factor for the absorption of SRF (*p* < 0.05, Table 3). 

**Figure 3:**
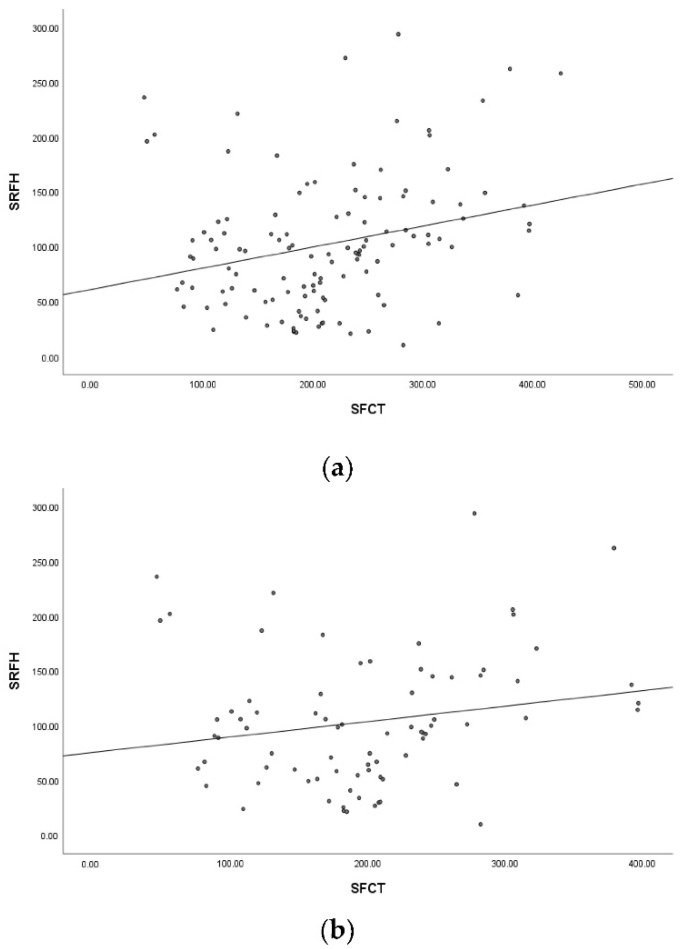

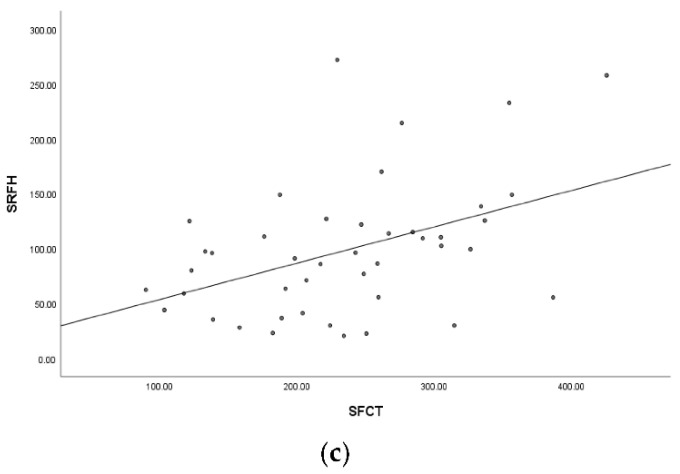
The scatterplots show the relationship between subretinal fluid height (SRFH) and subfoveal choroidal thickness (SFCT). (**a**): There is a weak positive correlation between SRFH and SFCT (r_s_ = 0.275, *p* = 0.002). (**b**): In the high myopia group, SRFH has no significant relationship with SFCT (*p* = 0.064). (**c**): In the non-high myopia group, SRFH is moderately positively correlated with SFCT (r_s_ = 0.402, *p* = 0.008).

The average SFCT in the high myopia group was significantly lower than that in the non-high myopia group (197.98 ± 81.28 vs 247.13 ± 84.39, *p* < 0.05). The SFCT and SRFH were positively correlated (r_s_ = 0.275, *p* = 0.002, Figure 3a). In the high myopia group, SRFH had no significant correlation with SFCT (*p* = 0.064, Figure 3b); in the non-high myopia group, SRFH was moderately positively correlated with SFCT (r_s_ = 0.402, *p* = 0.008, Figure 3c). CCFD in macular fovea with and without SRF was significantly different (0.63 ± 0.05% vs. 0.66 ± 0.05%, *p* < 0.05).

## 4. Discussion

Persistent SRF is common after successful SB surgery for primary RRD [6,7]. Among the 90 RRD eyes investigated in this study, 43 eyes (47.78%) had persistent SRF 3 months after SB. Choroidal capillary blood flow density is one of the main factors affecting SRF absorption. Our data show a significant difference in CCFD with and without SRF; CCFD without SRF was significantly higher than that with SRF, which is consistent with our previous research [8]. Choroidal capillary blood flow can promote the absorption of SRF; when CCFD decreases, the absorption of SRF slows down.

It has been reported that age is another major factor leading to persistent SRF. This and other research have shown that younger patients have a higher probability of delayed SRF absorption after SB [9,10]. Michael et al. [1] explored the factors of persistent SRF after PPV, and the results showed that high myopia could lead to delayed absorption of SRF, while younger age was not associated with persistent SRF. They considered that the thinning and retrogradation of RPE and atrophy of the choroid in high myopia might lead to a decrease in blood pumping function, thus affecting SRF absorption. Other studies have shown that axial length has no significant effect, while age is an important prognostic factor in the success rate of a single surgery [11]. Our results showed no significant difference in SRF absorption in high myopia, but there were significant differences between different age groups, suggesting that high myopia had no significant effect on SRF absorption, but age was a significant factor affecting the SRF absorption. We consider that the vitreous state may be a significant factor affecting SRF absorption. The vitreous body of young people is less liquefied and can thus play the role of biological packing, blocking the liquid channel and reducing the SRF absorption rate, resulting in persistent SRF. After PPV, this effect disappeared. This was also supported by the association between intraoperative drainage retinotomy and a lower incidence of persistent SRF [1]. The dysfunction of RPE in high myopia leads to delayed absorption of SRF. At the same time, a certain degree of vitreous liquefaction occurs in high myopia, which compensates for the effect of the decrease in choroidal blood density on the absorption of SRF to a certain extent. Therefore, our results showed that high myopia has no significant effect on the absorption of SRF after SB.

High myopia is different from low myopia. Pauline et al. [12] examined choroidal capillary blood in low myopia, and the results showed no significant correlation between choroidal capillary blood flow and the ocular axis and choroidal thickness in the macular fovea. This may indicate that the choroidal capillary layer has a compensatory mechanism for choroidal thinning in patients with low myopia. However, choroidal capillary flow density decreases in high myopia, which is related to an increase in the ocular axis and age [13]. Studies have found that high myopia can reduce the density of choroidal blood vessels, resulting in choroidal hypoxia and atrophy, leading to choroidal thinning [14]. In terms of choroidal blood flow, some studies have revealed that the density of large and medium-size choroidal vessels and choroidal capillaries in high myopia is lower than that in normal eyes [15,16]. Another study also found that the missing area of choroidal capillary blood flow in high myopia was larger than in normal eyes, the diameter and density of the choroidal capillaries in high myopia decreased, and the distance between adjacent capillary networks increased, resulting in a decrease in capillary blood flow density [17]. The decrease in CCFD leads to a reduction in the choroidal pump function, thus slowing down the SRF absorption.

Since the CCFD in high myopia was lower than that in non-high myopia, this may theoretically lead to delayed SRF absorption. Previous studies have found that SRF absorption in eyes with high myopia after PPV is delayed [1]. However, our results showed that high myopia was not related to delayed SRF absorption, which is consistent with our clinical observations. There was no significant difference in SRF absorption in patients with RRD after SB between the high and non-high myopia groups, indicating that other factors affected SRF absorption in patients with high myopia.

The results of this study showed a weak positive relationship between SRFH and SFCT; the thicker the choroid in the central fovea, the higher the height of the subretinal fluid. Therefore, the thickening of the choroid may lead to delayed SRF absorption. However, in the high myopia group, the SRFH was not significantly correlated with the SFCT (*p* = 0.064), and in the non-high myopia group, the SRFH was moderately positively correlated with the SFCT (r_s_ = 0.402, *p* = 0.008). Previous studies, including our studies, have shown that the ocular axis is closely related to the thickness of the choroid, the ocular axis of eyes with high myopia is longer, and the choroid is thinner than that of the non-high myopic eye [18,19,20]. In high myopia, dilatation of the eyeball can cause disproportionate choroidal thinning, with the most severe thinning occurring in the central fovea [21]. Some studies have suggested that choroidal thinning in high myopia is related to the loss of large blood vessels and the thinning of choroidal capillaries [22], while other studies concluded that atrophy of the middle choroid is also the cause of choroidal thinning in high myopia [23]. However, our results showed that preoperative thinning of the choroid due to high myopia did not affect the absorption of SRF. Some studies have found that the choroid is significantly thickened after SB surgery, which is considered to lead to choroidal blood stasis and an increase in the choroidal vascular pressure, resulting in choroidal hyperpermeability and choroidal vascular dilatation [24,25]. Therefore, we considered that the effect of surgery on choroid is much greater than that of high myopia. Eliott et al. [6] investigated the association between SFCT and persistent SRF after surgery for RRD, and the results showed that eyes with persistent SRF had significantly thicker SFCT than eyes without SRF. Multiple logistic regression analysis showed that SFCT of 280 μm or more was a significant factor associated with the persistence of SRF.

In addition, the high degree of vitreous liquefaction in high myopia may affect SRF absorption. Myopia is another age-independent risk factor for accelerated posterior vitreous detachment [26]. In high myopic eyes, posterior sclera dilation, vitreous volume increase, and vitreous degeneration and liquefaction occur earlier [27]. This myopic vitreous liquefaction can be explained by an increase in vitreous volume, which exceeds the production of coagulation components that fill the expansion chamber [5]. The concentration of collagen and hyaluronic acid in the vitreous of eyes with high myopia is 30% lower than that in normal eyes, and the degree of polymerization is also lower, especially in the central part of the vitreous cavity [28]. Hyaluronic acid can inhibit the phagocytic activity of RPE and slow down the absorption of liquid [13]. The hyaluronic acid level in the vitreous of high myopia is reduced and accelerates SRF absorption. Persistent SRF is less likely to occur after PPV than SB [29], which also suggests that the vitreous state may affect SRF absorption. A vitreous body with less liquefaction can play the role of biological packing, blocking the liquid channel, and reducing the SRF absorption rate [11]. Therefore, we consider that vitreous liquefaction in high myopic eyes accelerates the SRF absorption after SB, and vitreous liquefaction compensates for the effect of decreased CCFD on SRF absorption to a certain extent. Our research showed that high myopia had no significant effect on SRF absorption after SB surgery.

This study has several limitations. First, some patients were not regularly followed up with, resulting in a lack of data. Most of the patients without continuous follow-up had good SRF absorption and a low degree of myopia and, therefore, have little impact on the follow-up study. Second, the sample size was small, and the data were unevenly distributed, which might have deviated from the results. The population of RRD patients was small and difficult for long-term follow-up. Subsequent studies can further collect medical records pertinently, expand the sample size, and optimize grouping. Third, in addition to OCT and OCTA, fundus spontaneous fluorescence, FFA, ICGA, and other examinations could also be used for a comprehensive evaluation of choroidal function.

## 5. Conclusions

High myopia had no significant effect on SRF absorption after SB. The SRF absorption after SB surgery was related to the blood flow density of the choroidal capillaries. Age and vitreous state may be the main influencing factors of SRF absorption. The liquefied vitreous may compensate for the effect of decreased CCFD on SRF absorption in highly myopic eyes.

## Figures and Tables

**Table 1:** General information of patients.

Characteristic	*n* (%)
Sex	
Male	42 (46.67)
Female	48 (53.33)
Age	
<35 years	37 (41.11)
≥35 years	53 (58.89)
High myopia	
Yes	46 (51.11)
No	44 (48.89)
Absorption time of SRF	
≤3 months	47 (52.22)
>3 months	43 (47.78)

SRF: subretinal fluid.

**Table 2:** Fisher’s exact test results of high myopia and age for SRF absorption.

	Absorbed	Delayed Absorption	X^2^	*p*
High myopia			0.729	0.408
Yes	22 (47.83)	24 (52.17)		
No	25 (56.82)	19 (43.18)		
Age			5.210	0.032
<35 years	14 (37.84)	23 (62.16)		
≥35 years	33 (62.26)	20 (37.74)		

SRF: subretinal fluid.

**Table 3:** Binary logistics regression analysis of high myopia and age for SRF absorption.

	β	Wald X^2^	Sig	OR (95% CI)
High myopia	0.010	0.000	0.983	1.010 (0.404–2.523)
Age	0.993	4.384	0.036	2.701 (1.066–6.844)

SRF: subretinal fluid; OR: odds ratio; CI: confidence interval.

## Data Availability

The data generated or analyzed to support the findings of this study are available from the corresponding author on reasonable request.

## References

[1] Mimouni M., Jaouni T., Ben-Yair M., Almus S., Derman L., Ehrenberg S., Almeida D., Barak Y., Zayit-Soudry S., Averbukh E. (2020). Persistent loculated subretinal fluid after rhegmatogenous retinal detachment surgery. Retina.

[2] Kim Y., Woo S., Park K., Yu Y., Chung H. (2010). Comparison of persistent submacular fluid in vitrectomy and scleral buckle surgery for macula-involving retinal detachment. Am. J. Ophthalmol..

[3] Huang C., Fu T., Zhang T., Wu X., Ji Q., Tan R. (2013). Scleral buckling versus vitrectomy for macula-off rhegmatogenous retinal detachment as accessed with spectral-domain optical coherence tomography: A retrospective observational case series. BMC Ophthalmol..

[4] Mitry D., Charteris D., Fleck B., Campbell H., Singh J. (2010). The epidemiology of rhegmatogenous retinal detachment: Geographical variation and clinical associations. Br. J. Ophthalmol..

[5] Kim M., Park S., Park K., Woo S. (2019). Different Mechanistic Association of Myopia with Rhegmatogenous Retinal Detachment between Young and Elderly Patients. BioMed Res. Int..

[6] Chantarasorn Y., Oellers P., Eliott D. (2019). Choroidal Thickness Is Associated with Delayed Subretinal Fluid Absorption after Rhegmatogenous Retinal Detachment Surgery. Ophthalmol. Retin..

[7] Benson S., Schlottmann P., Bunce C., Xing W., Charteris D. (2007). Optical coherence tomography analysis of the macula after scleral buckle surgery for retinal detachment. Ophthalmology.

[8] Long K., Meng Y., Chen J., Luo J. (2021). Multifactor analysis of delayed absorption of subretinal fluid after scleral buckling surgery. BMC Ophthalmol..

[9] Kim Y., Ahn J., Woo S., Hwang D., Park K. (2014). Multiple subretinal fluid blebs after successful retinal detachment surgery: Incidence, risk factors, and presumed pathophysiology. Am. J. Ophthalmol..

[10] Abouzeid H., Becker K., Holz F., Wolfensberger T. (2009). Submacular fluid after encircling buckle surgery for inferior macula-off retinal detachment in young patients. Acta Ophthalmol..

[11] Park S., Lee J., Lee J. (2018). Scleral buckling in the management of rhegmatogenous retinal detachment: Patient selection and perspectives. Clin. Ophthalmol..

[12] Scherm P., Pettenkofer M., Maier M., Lohmann C., Feucht N. (2019). Choriocapillary Blood Flow in Myopic Subjects Measured With OCT Angiography. Ophthalmic Surg. Lasers Imaging Retin..

[13] Gregory C., Converse C., Foulds W. (1990). Effect of glycoconjugates on rod outer segment phagocytosis by retinal pigment epithelial explants in vitro assessed by a specific double radioimmunoassay procedure. Curr. Eye Res..

[14] Luu C., Lau A., Lee S. (2006). Multifocal electroretinogram in adults and children with myopia. Arch. Ophthalmol..

[15] Chan S., Wang Q., Wei W., Jonas J. (2016). Optical coherence tomographic angiography in central serous chorioretinopathy. Retina.

[16] Pang C., Sarraf D., Freund K. (2015). Extreme choroidal thinning in high myopia. Retina.

[17] Wong C., Teo Y., Tsai S., Ting S., Yeo Y., Wong W., Lee S., Wong T., Cheung C. (2019). Characterization of the choroidal vasculature in myopic maculopathy with optical coherence tomographic angiography. Retina.

[18] Teberik K., Kaya M. (2017). Retinal and Choroidal Thickness in Patients with High Myopia without Maculopathy. Pak. J. Med. Sci..

[19] El-Shazly A., Farweez Y., ElSebaay M., El-Zawahry W. (2017). Correlation between choroidal thickness and degree of myopia assessed with enhanced depth imaging optical coherence tomography. Eur. J. Ophthalmol..

[20] Ho M., Liu D.T., Chan V.C., Lam D.S. (2013). Choroidal thickness measurement in myopic eyes by enhanced depth optical coherence tomography. Ophthalmology.

[21] Ikuno Y., Tano Y. (2009). Retinal and choroidal biometry in highly myopic eyes with spectral-domain optical coherence tomography. Investig. Ophthalmol. Vis. Sci..

[22] Jonas J., Wang Y., Dong L., Guo Y., Panda-Jonas S. (2020). Advances in myopia research anatomical findings in highly myopic eyes. Eye Vis..

[23] Alshareef R., Khuthaila M., Januwada M., Goud A., Ferrara D., Chhablani J. (2017). Choroidal vascular analysis in myopic eyes: Evidence of foveal medium vessel layer thinning. Int. J. Retin. Vitr..

[24] Kim J., Lee E., Cho G., Bae K., Lee J., Han G., Kang S. (2017). Delayed Absorption of Subretinal Fluid after Retinal Reattachment Surgery and Associated Choroidal Features. Korean J. Ophthalmol..

[25] Lee J., Lee S., Kim H., Lee C. (2019). Comparison of short-term efficacy between oral spironolactone treatment and photodynamic therapy for the treatment of nonresolving central serous chorioretinopathy. Retina.

[26] Nguyen J., Nguyen-Cuu J., Mamou J., Routledge B., Yee K., Sebag J. (2021). Vitreous Structure and Visual Function in Myopic Vitreopathy Causing Vision-Degrading Myodesopsia. Am. J. Ophthalmol..

[27] Holekamp N., Harocopos G., Shui Y., Beebe D. (2008). Myopia and axial length contribute to vitreous liquefaction and nuclear cataract. Arch. Ophthalmol..

[28] Berman E., Michaelson I. (1964). The chemical composition of the human vitreous body as related to age and myopia. Exp. Eye Res..

[29] Fu Y., Chen S., Gu Z., Zhang Y., Li L., Yang N. (2020). Natural history of persistent subretinal fluid following the successful repair of rhegmatogenous retinal detachment. Int. J. Ophthalmol..

